# Rational Drug Design of Peptide-Based Therapies for Sickle Cell Disease

**DOI:** 10.3390/molecules24244551

**Published:** 2019-12-12

**Authors:** Olujide O. Olubiyi, Maryam O. Olagunju, Birgit Strodel

**Affiliations:** 1 Institute of Complex Systems: Structural Biochemistry, Forschungszentrum Jülich, 52425 Jülich, Germany; m.olagunju@fz-juelich.de (M.O.O.); b.strodel@fz-juelich.de (B.S.); 2 Department of Pharmaceutical Chemistry, Faculty of Pharmacy, Obafemi Awolowo University, Ile-Ife 220282, Nigeria; 3 Institute of Theoretical and Computational Chemistry, Heinrich Heine University Düsseldorf, Universitätsstraße 1, 40225 Düsseldorf, Germany

**Keywords:** hemoglobin, polymerization inhibitors, hemoglobin modifiers, sickle cell treatment

## Abstract

Sickle cell disease (SCD) is a group of inherited disorders affecting red blood cells, which is caused by a single mutation that results in substitution of the amino acid valine for glutamic acid in the sixth position of the β-globin chain of hemoglobin. These mutant hemoglobin molecules, called hemoglobin S, can polymerize upon deoxygenation, causing erythrocytes to adopt a sickled form and to suffer hemolysis and vaso-occlusion. Until recently, only two drug therapies for SCD, which do not even fully address the manifestations of SCD, were approved by the United States (US) Food and Drug Administration. A third treatment was newly approved, while a monoclonal antibody preventing vaso-occlusive crises is also now available. The complex nature of SCD manifestations provides multiple critical points where drug discovery efforts can be and have been directed. These notwithstanding, the need for new therapeutic approaches remains high and one of the recent efforts includes developments aimed at inhibiting the polymerization of hemoglobin S. This review focuses on anti-sickling approaches using peptide-based inhibitors, ranging from individual amino acid dipeptides investigated 30–40 years ago up to more promising 12- and 15-mers under consideration in recent years.

## 1. Introduction

Drug treatment of diseases follows different disease-dependent therapeutic strategies, such as replacing certain systemic deficiencies as seen in insulin management of type I diabetes mellitus, prophylactic treatments as seen in the use of pyrimethamine in preventing malaria, modulating receptor interaction often involving dysfunctional biochemical processes either in the host or in the offending pathogen. In a number of diseases of special interest, however, pathological sequelae as well as clinical manifestations can be directly traced to critical defects in protein folding and aggregation [1]. A number of diseases fall into this category, including Alzheimer’s disease, Parkinson’s diseases, Creutzfeldt–Jakob disease, type II diabetes mellitus, and sickle cell disease. Their clinical presentations can often be traced to mutational changes in amino acid sequence, which frequently instigate abnormal folding and aggregation behavior of the concerned protein. In sickle cell disease (SCD), a point mutation involving the replacement of glutamic acid at position 6 of the β-globin chain of hemoglobin to valine leads to the polymerization of hemoglobin [2]. In manifestation, SCD represents a symptom complex that involves dehydration of the Glu6 to Val6 mutated hemoglobin, which is called sickle hemoglobin or hemoglobin S (HbS), and elevated 2,3-diphosphoglycerate (2,3-DPG) levels whose interaction with hemoglobin reduces HbS solubility and promotes polymerization, also called sickling [3,4]. This ultimately leads to hampered O_2_ binding and transport, impaired erythrocyte morphology and interaction with endothelial surfaces [5,6], premature erythrocyte rupture and anemia, painful vaso-occlusive crisis, a general poor health, and, in many cases, death [7,8,9,10,11]. 

Despite growing understanding of the polymerization of HbS and its effects on red blood cells (RBCs), until very recently, only two drugs—hydroxyurea and L-glutamine—were approved by the United States (US) Food and Drug Administration (FDA) for the management of SCD [12]. Hydroxyurea is the most widely employed drug treatment of sickle cell anemia in different age groups [13,14,15,16,17,18]. While its clinically observed efficacy has been attributed to different effects at the cellular level [19], the most important mechanism of action relates to its ability to induce the production of fetal hemoglobin (HbF), which does not polymerize, and to increase the total concentration of hemoglobin [20,21]. Hydroxyurea remains a viable treatment option for SCD, and the concern of toxicities associated with its administration has largely been limited to side effects that resolve with medication discontinuation [22,23,24,25,26]. There have, however, been certain reports of associated malignancies [27,28,29,30,31,32], but further investigations are needed to categorically confirm these [33]. L-glutamine is the second approved drug treatment [12,34]. While its mechanism of action is not known, and only suggested to involve a reduction of oxidative stress via elevation of the levels of reduced glutathione [35,36], it is clear that it has no effect on hemoglobin S aggregation and hemoglobin production [37,38,39,40,41]. A third option for the treatment of SCD is hemopoietic stem transplantation, but its general applicability is limited by technical and cost considerations, and thus, out of the reach of SCD sufferers in third-world countries [42,43,44,45,46,47]. Of the millions of people with SCD, more than 75% are believed to live in Nigeria, Democratic Republic of Congo, and India [5,48,49]. These countries are additionally responsible for about 80% of global newborns having the causative Glu6 to Val6 mutation [50]. 

Recent reviews described different treatment modalities and efforts to develop new drugs targeting SCD [12,51,52]. A number of research attempts have been made to design interventions aimed at modulating the structural properties, aggregation tendencies, and defective O_2_ transport properties of sickle hemoglobin. For example, allosteric modulators and covalent modifiers of HbS that stabilize the non-polymer forming R-state Hb conformation have been reported and include the recently FDA approved voxelotor (GBT 440) [53] and derivatives of vanillin [54,55]. Compounds like senicapoc, a Gardos channel blocker, were also reported with the ability to prevent RBC dehydration [56]; clinical assessment in SCD, however, failed to find a correlation between improvements in hemolysis and vaso-occlusive crisis [57]. Selective inhibition of phosphodiesterase 9A by IMR-687 was recently reported to reduce both sickling and vaso-occlusion, which is believed to result from the induction of cGMP (cyclic guanosine monophosphate) and HbF [58]. Compounds which directly interact with HbS and disrupt the intermolecular contacts crucial to HbS polymerization have also been investigated, and they include small organic compounds [55], amino acid-based compounds (discussed in this review), as well as herbal preparations (e.g., Nix-0699 [59,60]). Other drug discovery efforts have focused on biochemical processes downstream of HbS polymerization rather than seeking to explore specific peculiarities of the aggregation process. A recent review by Eaton and Bunn argued in favor of research attention directed at the HbS polymerization process, especially because the aggregation kinetics as well as the circulatory transit time make it possible to achieve clinical improvement with only a small fraction of HbS aggregation inhibited [61,62]. Here, we review therapeutic approaches based on peptide-based drugs targeting the process of HbS polymerization. While this represents a departure from the traditional focus on small molecule inhibitors, especially covalent modulators of hemoglobin, for other protein aggregation diseases, peptide inhibitors turned out to be promising candidates for blocking the detrimental protein−protein interactions. Thus, this class of inhibitors deserves a closer inspection for their possible potential to treat SCD. An important aspect in this context is their larger size compared to traditional small molecular inhibitors, which, in principle, should translate into a greater interaction with polymerizing HbS and thus better antisickling potency. Before examining the role and potential of peptide inhibitors in SCD, we first provide a brief overview of the structure and aggregation of sickle hemoglobin, as well as of previous therapeutic approaches aimed at inhibiting HbS polymerization, allowing peptide inhibitors to be put into context.

## 2. Hemoglobins: Structure, Function, and Aggregation

**Figure 1:**
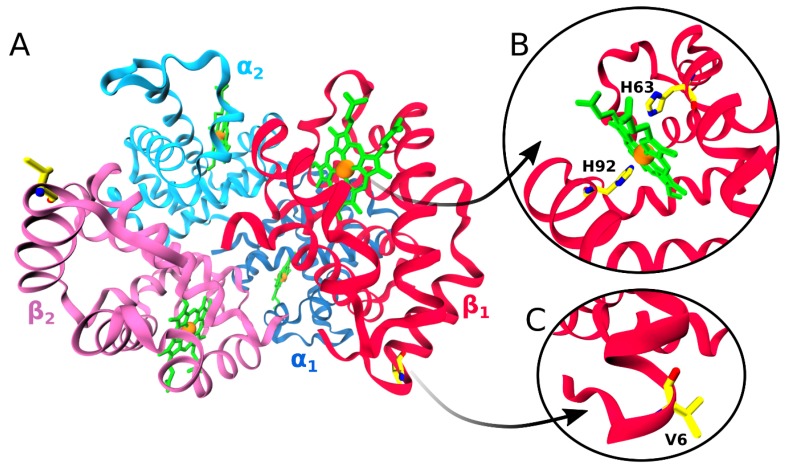
(**A**) The quaternary structure of HbS consisting of two α subunits (here denoted α_1_ and α_2_ for ease of distinction, shown in shades of blue) and two β (β_1_ and β_2_, shades of red) subunits. Each globin subunit carries one heme (green), including an Fe^2+^ ion (orange). (**B**) The hemes are linked to the globin by covalent bonds between their irons and N_ε_ of histidines His87 of the α chains and His92 of the β chains, known as the proximal histidines. On the other side of the hemes, the distal histidines are located, which are His58 in the α chains and His63 in the β chains. (**C**) The single mutation Glu6Val happens on the surface of the β chains near their N-terminus. The His and Val residues are shown as sticks and are colored by atom name (C: Yellow; N: Blue; O: Red). This figure was produced using PDB entry 5E6E [63].

The function of the red blood cells and their hemoglobin is to carry oxygen (O_2_) from the lungs to all the body tissues and to carry carbon dioxide (CO_2_) back to the lungs. This function is enabled by the structural characteristics of hemoglobin (Hb), allowing it to bind O_2_ and CO_2_. Both HbA, which refers to the wild-type hemoglobin present in individuals without sickling disorder, and HbS exist as tetramers consisting of two α subunits and two β subunits arranged into a pseudotetrahedral symmetry (Figure 1A). With the two 141-residue α-globin chains and the two 146-residue β-globin chains, and each globin chain carrying one heme group, the full HbA/HbS assembly contains 574 amino acids and four heme molecules. It is from these four heme molecules and the four globin chains that hemoglobin derives its name. 

The quaternary structure of hemoglobin is maintained by relatively weak but precisely coordinated non-covalent interaction forces, including van der Waals interactions, hydrogen bonds, and salt bridges between the different globin chains. In total, there are 30 helices in the hemoglobin structure: The two α-globin chains feature a total of 14 helices between them, while the β-globins have 16 helices. Each globin chain is covalently linked to a heme molecule via their proximal histidine residue (His87 in the α-globin chains and His92 in the β-globin chains). The heme, in turn, consists of a protoporphyrin part and a centrally coordinated iron ion (Figure 1B). The local environment of the globin molecules maintains the coordinated iron ion in its reduced form, in which state it can form a total of six bonds. Four of the six coordination sites of the ferrous ion are covalently bonded to the protoporphyrin ring, another to the imidazole side chain of the histidine residues, while the sixth coordination site allows for binding and unbinding of dissolved gases. It is this last coordination site that is responsible for O_2_ binding. Following Fe^2+^ binding, bound oxygen establishes hydrogen bonding with the imidazole side chain of His58 in the α-globins, and His63 of the β-globins, the distal histidine. In this state, heme adopts a relaxed, conformationally unstrained arrangement structurally representing the “R” conformation and functionally the oxygenated hemoglobin [63]. 

**Figure 2:**
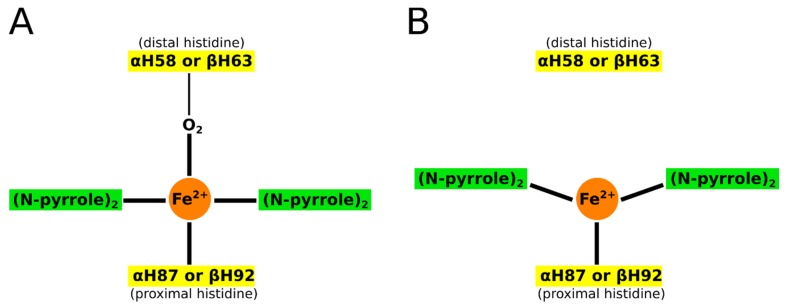
Schematic representation of the main structural differences between the (**A**) R and (**B**) T conformations of hemoglobin.

In the deoxygenated form, the distal histidine side chains have a propensity to swing out of the heme pocket, thus allowing a compression of the surrounding helices with respect to each other, which in turn causes Fe^2+^ to move out of the porphyrin plane [64]. This gives rise to a tensed conformation (“T” conformation) with the heme adopting a dome-like arrangement (Figure 2). This structural change precipitates a series of further changes in the remaining body of the HbS protein, which, under deoxygenation and dehydration conditions, provokes a pathologic cascade that ultimately leads to clinical manifestations.

**Figure 3:**
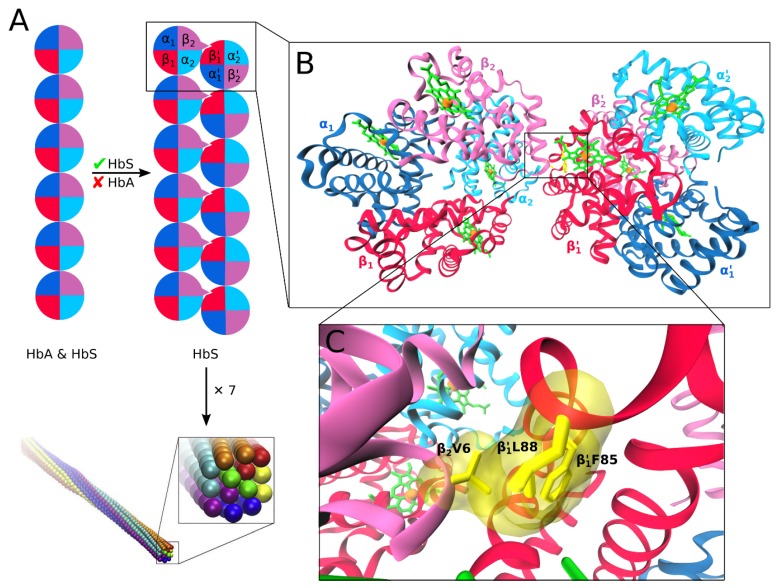
(**A**) Schematic representation of how the Glu6Val mutation modifies normal hemoglobin polymerization of HbS heterotetramers, involving linear Hb aggregates formed by both HbA and HbS (left) into double HbS filaments (right). The hemoglobin tetramer is represented as a circle, such that one quarter corresponds to one protein subunit using the same coloring as in Figure 1. The βGlu6Val mutation is indicated as a protrusion from the circle in the β2 subunit and the hydrophobic pocket as a nick in the neighboring $\beta_{1}^{\prime }$ subunit. Seven double filaments aggregate further to form fibers (bottom, reproduced with permission from reference [83]). (**B**) A dimer formed by two HbS aggregates is shown. (**C**) This aggregation is mediated by β_2_Val6 interacting with the hydrophobic pocket formed by $\beta_{1}^{\prime }$Phe85 and $\beta_{1}^{\prime }$Leu88. The side chains of these three residues are shown as yellow sticks and also transparent van der Waals surfaces to better indicate the space these residues occupy. Panels B and C were produced from PDB entry 2HBS [84].

It deserves noting that the Glu6Val mutation involves an amino acid replacement at the HbS surface (Figure 1C) and, as such, only affects protein–protein interaction involving surface residues [65,66,67,68,69,70,71,72,73,74,75,76,77,78], without any effect on amino acids located at the core [69,70]. The side chain of Val6 in the β-globin structure (the donor β-globin) of HbS forms a hydrophobic key, which fits well into an essentially hydrophobic cavity formed by Phe85 and Leu88 of the β-globin of an adjacent HbS molecule (Figure 3). It should be noted that both HbA and HbS form linear aggregates involving the formation of axial contacts between Hb molecules. Only in the case of HbS, these linear aggregates grow into double filaments, facilitated by lateral βVal6–β′Phe85/β′Leu88 contacts (where the prime indicates that Phe85 and Leu88 belong to another hemoglobin than Val6). The double filaments further assemble into ~200 Å thick fibers, which eventually accumulate in highly complex, pathological HbS fiber networks [79]. These aggregates affect the functionality of the red blood cell by destroying their structural pliability into stiffened and deformed erythrocytes. Differences at the cellular level, for instance, originating from different degrees of cellular dehydration or oxidative stress, may further complicate the HbS polymerization, such that each patient’s clinical manifestations are, to some extent, unique [80,81,82]. 

## 3. HbS as a Target for Drug Design

### 3.1. HbS Aggregation Is An Inefficient Process

Efforts to rationally design antisickling agents have often viewed the sickle hemoglobin both as the drug target as well as the starting point for lead discovery. Such efforts are indeed not new; the 1970s through the 1980s witnessed a good deal of research interest into the molecular nature of the HbS molecule, as well as the search for compounds capable of disrupting its polymerization. A prevailing doubt about the suitability of the HbS molecule as target for drug development has to do with the perceived limitation imposed by its high content level in man (about 450g) [61], suggesting that an intolerably high dose of antisickling compound would be required to achieve clinically useful degrees of inhibition [85]. This perception was mostly based on an aggregation model built on the assumption of a highly efficient nucleation dependent HbS polymerization process believed to involve two nucleation stages, beginning with a rate-limiting homogeneous nucleation, followed by a highly efficient heterogeneous nucleation phase [86,87]. For aggregation to occur, the delay time associated with the homogeneous nucleation should necessarily be shorter than re-oxygenation circulation time, which is the time required for the hemoglobin to pass through the blood vessels and be re-oxygenated [88]. In light of recent findings [89,90], there is increasing need to revisit what is accepted with respect to HbS polymerization kinetics. In a recent study employing high resolution differential interference contrast (DIC) microscopy (55 nm resolution at 1 Hz, the highest resolution currently available for HbS aggregation kinetics), monomer incorporation into HbS polymers was found to be a highly inefficient process, with only 30,000 out of one million HbS monomers incorporated per second [90]. This translates to a 3% efficiency for HbS polymerization as against the previously reported monomer incorporation efficiency of more than 95% [91,92]. This observation is supported by the finding of Wang and Ferrone, who, based on light scattering experiments, revealed that the overall thermodynamics into double filaments (Figure 3A) is marginally unfavorable, with the axial contacts being 1.8 kcal/mol weaker than the lateral contacts [93]. At such a low polymerization efficiency, HbS monomer binding and unbinding events are only marginally in favor of polymer growth, such that small disturbances (for instance, resulting from inhibitor binding) are sufficient to push the equilibrium towards polymer disassembly. Castle et al. calculated the magnitude of binding disturbance required and estimated it to be a 1.2 kcal/mol change in HbS monomer−polymer interaction in 5% of the available HbS molecules that is required to halt the polymerization process (see reference [90] for the calculation). This agrees qualitatively with the earlier estimated ~1.5 kcal/mol hydrophobic free energy contribution resulting from Val6 binding within the Phe85/Leu88 pocket [94]. With about 30 picogram (pg) of hemoglobin per RBC [95,96], disruption of polymerization in less than 1.5 pg HbS per cell should in principle be sufficient to frustrate aggregation, especially considering that only between 40 and 60% of the RBCs typically undergo sickling [97]. This reasoning does not only bring HbS polymerization within the purview of non-covalent inhibition, but it also rationalizes why antisickling effects have been observed for various small molecular weight inhibitors [98,99,100]. For instance, screening for non-covalent antisickling agents that reverse HbS polymerization by altering RBC shape and volume (towards more spherical structures with larger volumes) discovered antisickling properties for gramicidin A and monensin A at concentrations of 200 pM and 2 nm, respectively [101]. Another example is the aggregation inhibition by HbF, which is required to be present in a just a little fraction (0.2) of total hemoglobin of SCD patients to achieve clinical resolution of symptoms [102,103]. This antisickling effect of HbF serves as the mechanistic basis for SCD treatment with HbF-inducing hydroxyurea (see Introduction). Like HbF, addition of HbA to polymerizing HbS has also been shown to inhibit HbS aggregation [104].

### 3.2. Antisickling Effect and HbS Conformation 

**Figure 4:**
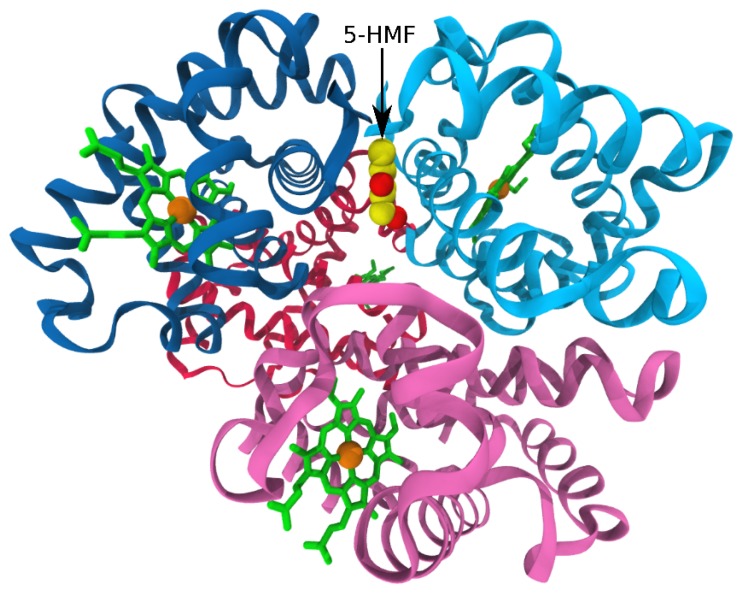
Binding of 5-hydroxymethylfurfural (5-HMF; yellow/red) in the α-cleft of HbS via hydrogen bonds and hydrophobic interactions formed with both α-globins, stabilizing the R-state conformation. The coloring scheme from Figure 1 is used for HbS. The figure was produced from PDB entry 5URC [98].

Targeting sickle hemoglobin for inhibitor design does not only aim to directly inhibit its aggregation into multi-stranded polymers, but also includes approaches that either result in the stabilization of the R conformation of the HbS molecule, or the destabilization of the T conformer [105,106]. Compounds whose antisickling properties are based on this concept include vanillin and pyridyl derivatives of vanillin, 5-hydroxymethylfurfural (5-HMF), and the recently approved voxelotor (GBT 440) [54,99,106,107,108,109,110]. They bind to the N-terminal valine (and possibly lysine) residues of the α-globin chains of HbS (Figure 4) [98], forming a reversible Schiff-base adduct which stabilizes the R-state and/or destabilizes the T-state, increasing hemoglobin solubility, and thus inhibiting HbS aggregation. Iqbal et al. employed an electrochemistry-based technique to investigate HbS polymerization in the presence of vanillin and 5-HMF [86]. At HbS concentrations of 100 mg/mL, aggregation inhibition was obtained for vanillin concentrations corresponding to 0.5:1, 1:1, and 10:1 mole ratios relative to HbS. A similar pattern was obtained for 5-HMF, except for an interesting observation that the 0.5:1 inhibitor/HbS ratio was found to slightly promote aggregation. At 1:1 inhibitor/HbS concentration, both compounds achieved roughly 70% aggregation inhibition, while a near perfect inhibition was recorded when the inhibitor concentration was increased to achieve a 10:1 mole ratio relative to the hemoglobin. In scanning the inhibitors against HbS, Iqbal et al. employed an HbS concentration that is about three orders of magnitude smaller than the intracellular concentration of hemoglobin, which is 334 mg/mL assuming an RBC volume of 90 fL and mean corpuscular hemoglobin of 30 pg. At such higher cellular content of hemoglobin, a more efficient system of inhibition is probably needed. Thus, continuing searches for antisickling agents is warranted, independent of the successful progression of GBT440 through phase III clinical trial leading to its recent FDA approval.

### 3.3. Antisickling Agents from In Silico Screening

**Figure 5:**
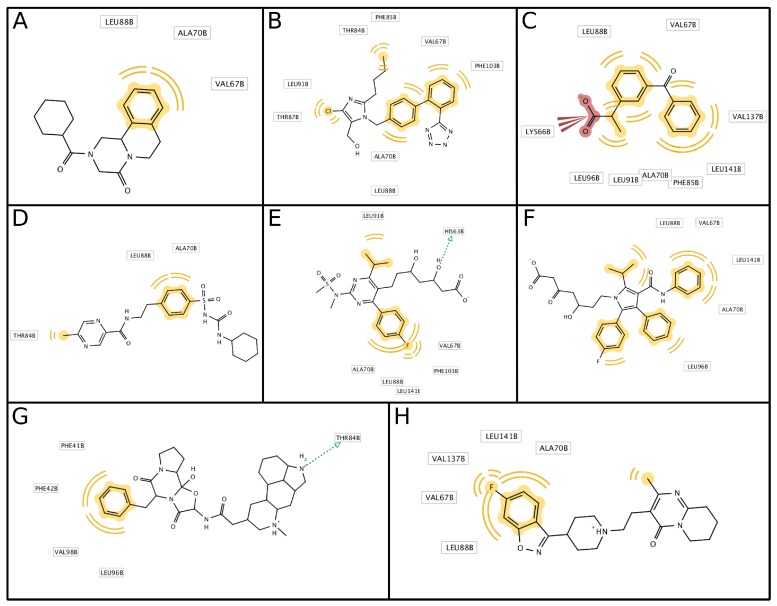
Computational models of the HbS interaction with compounds with antisickling activity from drug repurposing investigation in reference [111]: (**A**) praziquantel, (**B**) losartan, (**C**) ketoprofen, (**D**) glipizide, (**E**) rosuvastatin, (**F**) atorvastatin, (**G**) ergotamine, (**H**) risperidone. The orange interactions are of hydrophobic nature, preferentially with aromatic HbS residues, while the red color in panel C indicates an electrostatic interaction. Green arrows show hydrogen bonds.

Drug repurposing presents an attractive proposition to treat both common and rare diseases, considering the high attrition rates, substantial costs, and slow pace of new drug discovery and development. As drug repurposing involves the use of de-risked compounds, this approach should entail lower overall development costs and shorter development timelines. A first step towards drug repurposing for SCD was made in a recent computational screening of existing FDA approved drugs for their potential antisickling properties [111]. In this work, virtual screening was employed to screen existing drugs with the AutoDock Vina score, which were then rescored using an effective energy that specifically emphasizes the presence, size, and electronegativity of the chemical fragments present in each screened compound capable of competitively disrupting Val6 binding to the βPhe85/βLeu88 pocket. A number of compounds identified by this approach were shown in preliminary tests to possess antisickling activity. The concentrations of the compounds ranged from 0.02 M for glipizide, ketoprofen, and losartan to 2.2 M for atorvastatin. The docking-generated models in Figure 5 suggest that, in all but one case, binding of the compounds involves βPhe85 and/or βLeu88, which, however, should be considered with caution, given the lack of structural data of the HbS−ligand complexes. Interestingly, at least two of these drugs are given for adjunctive treatment of SCD because of their restorative effect against albuminuria in patients with SCD [112,113]. However, thus far, there is no other record reporting effects of these compounds in sickle RBC.

### 3.4. Interprotein Contacts During HbS Aggregation

In the quest to target HbS to directly disrupt polymerization as therapeutic approach, one should consider that this may be more challenging than it first seems because of the plethora of multiple binding sites that, when interfered with, may influence the conformational preferences of HbS that favor or disfavor polymerization. There exists a good number of data suggesting that both intra- and interpeptide contacts sponsor the polymerization process of HbS, which involves interactions at multiple sites on the hemoglobin molecule. Without doubt, the aberrant valine residue at position 6 of the β-globin is involved, believed to be in immediate contact with β′Phe85 and β′Leu88 (Figure 3). It is thought that concurrently, to this contact, a hydrogen bond between βThr4 and β’Asp73 is formed due to the spatial proximity between these residues. In addition to these primary contacts, secondary contacts, which involve hydrophobic and also a number of ionic interactions [84,88,114,115,116,117,118], have been identified and proposed to either influence directly the polymerization process, modulate the conformational equilibrium between the R and T state, or simply modify the solubility of deoxygenated HbS. For example, the αAsn78→Lys mutation leads to an increase in the solubility of deoxy-HbS, alleviating the severity of SCD [118,119]. Another challenge for the design of antisickling agents aimed at disrupting the aggregation process is a common problem when targeting protein−protein interactions, because these interaction sites are typically flat and large, quite different from the “grooves” or pockets in which small molecules typically bind. Peptides are ideal candidates to overcome this problem, as they can mimic a protein surface to effectively compete for binding [120]. In the following section, we present selected efforts to design peptides or peptide-based systems intended as inhibitors of HbS polymerization. For a review of non-peptide chemical classes of HbS polymerization inhibitors, the reader is referred to references [51,61,83] for excellent treatment of the topic. 

## 4. Amino Acid-Derived Antisickling Compounds

### 4.1. Peptide Length and HbS Polymerization Inhibition

One of the oldest ideas driving the design of HbS aggregation inhibitors relies on the acknowledgment of the causal role played by the Glu6Val β-globin mutation on disease development. Many of the earliest reported efforts sought to obtain compounds with the right combination of hydrophobicity, shape, and charge complementarity that, in principle, can bind within or in the immediate vicinity of the cavity formed by β′Phe85 and β′Leu88 and, at the same time, possess charged groups oriented outwards. This outward projection is to prevent βVal6 of an incoming β-globin chain from binding as part of the lateral contact in HbS polymer. While the nature of βVal6 binding site would seem to place an upper limit on the molecular size of prospective inhibitors capable of binding to this site, in reality, conflicting reports have been published by different groups working on amino acid-derived inhibitors. In the late 1970s and early 1980s, Rich and co-workers examined short peptide inhibitors (up to pentapeptides) of HbS aggregation based on the belief that amphipathic nature was required to inhibit the polymerization of deoxygenated HbS [121,122]. Out of the peptides examined, the lowest minimal inhibitor mole ratio (MIMR) of peptide to HbS necessary to prevent HbS polymerization was found for N-terminally succinylated (Phe)_3_, (Phe)_3_–Arg, and (Trp)_2_ (Table 1), where succinylation in each case served to enhance peptide solubility, or to modulate net charge, or both. It is, however, important to note that the concentrations of the peptides employed in these works were too high to be of any direct benefit in a clinical setting: The best inhibitory effects were achieved with peptide/HbS mole ratios of about 10. While structural data were lacking to categorically conclude on structure–activity relationship (SAR), the reported pattern of inhibition showed inhibitory activity increasing with peptide chain length. This could point to the fact that the nature of HbS−HbS interaction surface requires sufficiently large inhibitors to effectively disrupt crucial amino acid interactions. It is thus likely that more potent peptide inhibitors will be achieved with peptide lengths longer than those screened in these studies [121,122]. Interestingly, a similar trend was observed with peptide inhibitors of amyloid-β aggregation, whereby highly potent aggregation inhibitors were achieved with 12-amino acid peptides, while shorter ones lacked this property [123,124,125]. In fact, a phage display work by Hanson et al. in 2013 successful identified a highly potent 12-residue peptide (Hb-B10, sequence CHNLLPTPWWCA) with a micromolar range (21 µmol/L) binding affinity for hemoglobin [126]. Even though the intention was not to target HbS polymerization but to aid the clearance of circulating hemoglobin, the outcome of this research shows that indeed it is possible to obtain peptide-based systems with a HbS binding affinity required for clinical intervention.

The work of Kubota and Yang was similarly founded on the special importance of the βVal6 residue during HbS polymerization by designing oligopeptides to mimic the N-terminal segments of the β-globin chain of Hb [127]. The idea behind this approach is that such peptides would interact with the βPhe85/βLeu88 pocket, or any other complementary binding site, and thus inhibit HbS polymerization. The tested peptides were indeed found to exhibit significant HbS aggregation inhibitory attributes, with the β_1–6_ hexapeptides of the N-terminal end of both HbA (sequence VHLTPE) and HbS (sequence VHLTPV) molecules reported to increase the minimum gelling concentration (MGC) by about 75% [127]. The MGC is the concentration of HbS required to form a gel (or polymer), which is about 9.5 g/dL in the absence of peptide inhibitors, and an aggregation inhibitor is expected to increase this value. The highest inhibitory activities were obtained at peptide/heme mole ratios of between 2 and 2.5. Considering that there are four heme molecules per hemoglobin, this translates to a peptide/hemoglobin ratio of 8 to 10, which is in the MIMR range reported by Rich et al. [121,122] and listed in Table 1. These concentrations, like those reported in [121,122], are too high to have any clinical applicability. Truncating the length of the oligopeptides below six residues significantly reduced the inhibitory effect, which seems to suggest that the β_1–6_ hexapeptides might indeed interact with the βVal6 binding site on the β-globin chain [127]. According to the authors, hexapeptides, but not shorter oligopeptides, are likely to preserve the secondary structure necessary to provide the complementary shape needed to interact with the βVal6 binding site. The lack of structural data, however, makes this interpretation of the experimental outcome, at best, speculative; it is possible that the peptides interacted at other sites of the HbS molecule. Hexapeptides mimicking both HbA and HbS N-terminal segments produced similar inhibitory effects, while increasing the peptide length beyond six did not improve activity, although shorter peptides were less effective. Interestingly, in a separate work, it was observed that longer oligopeptide inhibitors involving sequences β_1–12_ (sequence VHLTPVEKSAVT), β_3–13_ (sequence LTPVEKSAVTA), β_4–8_ (sequence TPVEK), and β_4–10_ (sequence TPVEKSA) of HbS promote HbS polymerization [128], as they decrease the solubility of HbS [129]. 

The susceptible balance between peptide sequence, length, and structure for the capability to inhibit HbS polymerization is also demonstrated in a more recent work [130]. Akbar et al. studied the effects of 15-, 11-, 7-, and 3-mer peptides derived from one of the helices of the β-globin chain of hemoglobin. In the case of the 15-mer peptide, the sequence comprised the β-globin residues 65−79 with sequence KKVLGAFS[**H**/**L**]GLAHLD, where, at position 73, the β73His and β73Leu mutations were included instead of the native β73Asp, as, in HbS, these mutations were previously observed to inhibit HbS aggregation [131]. The shorter peptides with 3, 7, and 11 residues failed to inhibit polymerization, suggesting the importance of secondary structure and multiple contact points for the observed inhibitory activity. For the longer peptide, it was found that the β73His 15-mer peptide more significantly inhibited polymerization compared with the β73Leu 15-mer peptide. The β73His 15-mer peptide is believed to interact with β4Thr and thus disrupt the hydrogen bonding between β4Thr and β’73Asp, and also hydrophobic interactions involving β6Val due to its spatial proximity. However, it should be mentioned that a peptide/HbS ratio of 3:1 was needed to obtain a noteworthy delay in HbS polymerization [131]. While it is likely that different hemoglobin binding sites were employed by these peptides, they represent about 70% improvement in potency over the peptides studied in earlier works [121,122,127]. The outcomes of the different experiments suggest that there is no simple relationship between peptide length and HbS polymerization inhibition. Other factors that are important for inhibitory activity are considered in the following parts.

### 4.2. Peptide Hydrophobicity and Hydrophilicity

The effect of charged groups in designed peptide inhibitors of HbS polymerization has been somewhat difficult to generalize, with different works reporting both positive and negative inhibitory effects [111,115,121,122,126,128,132,133]. Some experiments involving large short-peptide libraries observed that the inhibitory effect depended more on the presence of hydrophobic rather than charged residues, and that peptides containing tryptophan [128,129] and phenylalanine [134] were found to display the highest polymerization inhibition. This also agrees with the results published by Rich et al. (Table 1) [121,122]. These seem to suggest that the HbS aggregation inhibitory effect of the studied peptides depended more on hydrophobic contacts formed with the hemoglobin than on specific charge interactions. 

**Figure 6:**
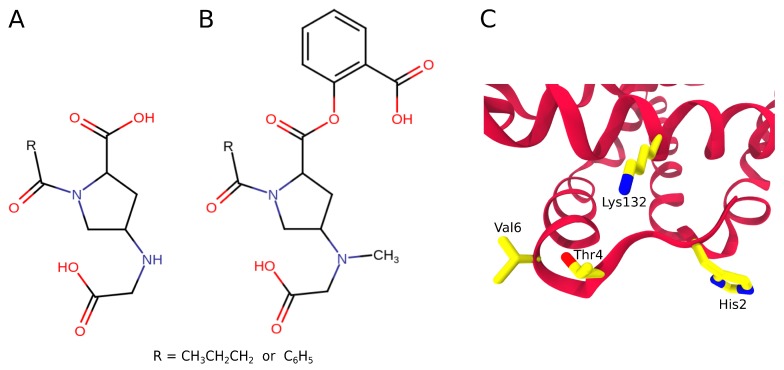
Proline derivatives tested for HbS anti-polymerization efficacy: (**A**) (4S)-1-butyryl-4-[(carboxymethyl)amino]-l-proline and (**B**) (4S)-1-butyryl-4-[(carboxymethyl)methylamino]-l-proline-2-ester with salicylic acid and their 1-benzoyl analogues [135]. (**C**) The anticipated binding site of these ligands is formed by βHis2, βThr4, and βLys132, which is thought to disturb the binding of βThr4 to β’Asp73 and of βVal6 to β’Phe85/β’Leu88. The salicylate group in (**B**) is expected to covalently bind to βLys132.

The work of Abraham et al. also endeavored to disrupt not only the βVal6−β’Phe85/β’Leu88 interaction, but also the hydrogen bonding between βThr4 and β’Asp73 [135]. To this end, four proline derivatives (Figure 6A,B) were developed: Two of them were designed to primarily form hydrogen bonds with His2, Thr4, and Lys132 of the HbS β-globin, while the other two were designed to bind covalently to βLys132, as well as to interact with βHis2 and βThr4 via ionic and hydrogen bonds. Based on the overall fold of the β-globin chain, it was expected that the peptides were placed enough in the vicinity of βVal6 to also enable the disruption of the βVal6−β’Phe85/β’Leu88 interaction (Figure 6C). Two of the compounds contain a salicylate moiety to allow for possible covalent interaction with βLys132, as salicylate and other aromatic esters have been reported to acetylate lysines of the Hb β-globin [136]. While the two non-covalently binding proline derivatives successfully inhibited polymerization of deoxygenated HbS, though with inhibitory levels of only a fraction of that of phenylalanine [134], the two compounds with a salicylate group were mildly aggregation-promoting. This observation is not totally unexpected, as aspirin and other salicylate esters had been known to promote sickling, and this effect must have been inherited from the salicylate group via a mechanism speculated to involve αArg141 acetylation and the consequential stabilization of the HbS T conformation [137]. The relatively low levels of inhibition reported for this set of peptides further suggests that hydrophobicity is more important for disrupting polymer contacts. This, however, should not downplay the role of ionic interactions in hemoglobin polymerization, which was observed to show pH and ionic concentration-dependency pointing at specific roles for ionic interactions in modulating aggregation [115,138]. 

### 4.3. Effects of Amino Acids and Specific Chemical Properties

**Figure 7:**
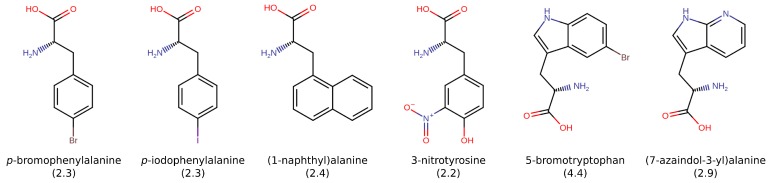
Phenylalanine, tyrosine, and tryptophan derivatives and analogues with significant anti-polymerization properties. The enhancement of the anti-polymerization capacity with respect to phenylalanine is given in parentheses [139].

Following the discovery of tryptophan and phenylalanine (tryptophan was found to possess a 2.1-fold higher anti-polymerization property of that of phenylalanine [139]) as the amino acids with the highest HbS polymerization inhibitory properties [121,122,128,129,134], efforts were expended to design analogues of the two amino acids with the aim of improving inhibitory efficiency [139,140]. Poillon studied 42 derivatives and analogues of alanine with varied aromatic side chains substituted at the β-carbon atom of alanine. Of all investigated derivatives and analogues, the six most potent aggregation inhibitors are shown in Figure 7. Again, the millimolar range inhibitory concentrations obtained (from 6 mM for 6-bromotryptophan to 30 mM for phenylalanine) are too unrealistic concentrations for clinical relevance. Other factors affecting the chemistry and hydrophobic nature were also believed to have contributed to the observed activities. Aromatic substitutions as opposed to aliphatic side chains, bicyclic aromatic rings as opposed to monocyclization, bromination compared with other halides, as well as 1-naphthyl substitution as opposed to 2-naphthyl substitution were observed to be associated with the highest HbS polymer destabilizing effect. Also, in 1977/1978, it was speculated that more efficient inhibitors might be achieved by enhancing the polarizability of the aromatic nucleus via appropriate substitution with heavy halogen or aryl groups [127,141]. This prediction is supported by the findings of Poillon as, for example, 5-bromotrytophan showed anti-polymerization activities roughly twice as effective as tryptophan and 4.4 times greater than that of phenylalanine [128]. In the absence of larger SAR studies that systematically vary each structural parameter, it is difficult to derive categorical conclusions from the published data. Nevertheless, it would seem that the peptide inhibition obtained by varying the structure (e.g., by aromatic substitution) was indirectly linked to changes in hydrophobic character, and better inhibitors (depending on the specific HbS sites targeted) will likely require a good degree of balance between different chemical properties of amino acids constituting the peptide inhibitors. 

**Figure 8:**
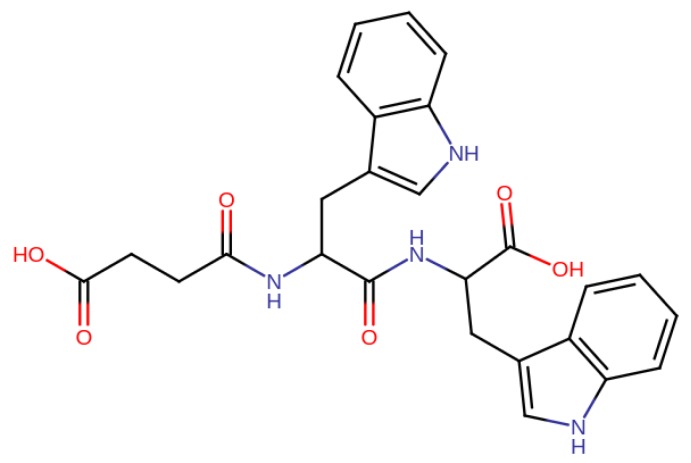
Succinyl-l-tryptophan-l-tryptophan (STT) increased the HbS solubility in a dose-dependent manner [79].

In 1986, Perutz et al. published their x-ray crystallography-based work on the binding between deoxygenated hemoglobin and four halogenated derivatives of aromatic oxyacetic acids (clofibric acid, ethracrynic acid, bezafibrate, *p*-bromobenzolyoxy acetic acid) and succinyl-l-tryptophan-l-tryptophan (STT) [79]. The objective of their work was to exploit stereo structural attributes of the HbS molecule in designing agents capable of inhibiting the process of HbS polymerization. The five studied compounds exhibited highly variable effects on HbS sickling ranging from HbS aggregation inhibition to the facilitation of HbS aggregation. These varied activities were believed to result from a significant degree of diversity existing in HbS binding sites employed by the different compounds. The short peptide STT (Figure 8) was observed to exert a dose-dependent increase in HbS solubility, the highest value being obtained at a 40 mM concentration. The low-resolution crystallographic data revealed that STT preferentially binds at the entrance to the central cavity between the two α-globins of one hemoglobin molecule via hydrogen bonds and several van der Waals interactions. This binding position is similar to the pose of 5-HMF (Figure 4), vanillin, and its pyridyl derivatives, but while these compounds stabilize the R state, STT only binds to the T state of hemoglobin. It was suggested that by increasing the solubility of deoxygenated HbS, STT inhibits polymerization and thus serves as a good starting point for drug design.

### 4.4. Highly Potent Peptide Inhibitors

**Figure 9:**
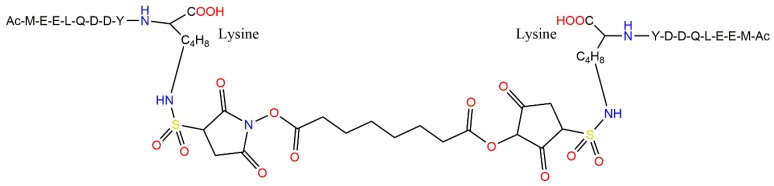
The structure of the highly potent 18-residue “mirror image” HbS polymerization inhibiting Peptide II featuring two Ac-MEELQDDYK units linked by a bis(sulfosuccinimidyl) suberate [142].

Motivated by reported binding of a fragment of the N-terminus of the erythrocyte band 3 protein to the 2,3-DPG (the endogenous allosteric effector that binds deoxygenated hemoglobin and stabilizes it in the T conformation) binding at the β-cleft of hemoglobin [142], Danish et al. in 1994 designed short peptides based on the band 3 protein and investigated their abilities to inhibit HbS polymerization [143]. Three peptides were studied: Peptide I N:1-15AA with sequence Ac-MEELQDDYEDDMEEN corresponding to the first fifteen amino acids of the band 3 protein, Peptide A N:1-8AA+K with sequence Ac-MEELQDDYK, and a “mirror image” Peptide II containing two Peptide A N:1-8AA+K units linked with a bis(sulfosuccinimidyl) suberate via the two N_ε_ atoms of the lysine side chains (Figure 9). Oxygen binding studies conducted in the absence of 2,3-DPG revealed a dose-dependent rightward shift mimicking 2,3-DPG binding for Peptide N:1-15AA and Peptide II, indirectly indicating interaction with the 2,3-DPG binding site, since all other factors were kept constant. While only marginal improvements were recorded in HbS solubility and polymerization assay for the shortest peptide, Peptide A N:1-8AA+K, Peptide I N:1-15AA, and Peptide II displayed significantly improved HbS solubility and polymerization inhibition profiles. The highest effects were observed for Peptide II. It is noteworthy that Peptide II at a peptide-to-hemoglobin concentration as low as 0.25:1 already significantly inhibited HbS polymerization, while also moderately increasing hemoglobin solubility. The highest inhibition of polymerization was observed at peptide/HbS concentration of 1:1. The suggested mode of action assumes that Peptide II would bind at the 2,3-DPG binding site, while the other end of the peptide would bind similarly to another deoxy-HbS molecule. The resulting ternary complexes (which were called “binary hemoglobin complexes” by Danish et al. [143]) would be incapable of forming HbS double filaments.

In summary, the degrees of HbS polymerization inhibition reported by the different groups vary widely and depend on multiple factors. To fully understand how these different factors influence activity, it is important to have carefully designed studies that systematically vary each of the structural variables. What we can learn from the experiments discussed in this review and from our understanding of the structural complexity of the hemoglobin molecule is that it is likely that different peptide inhibitors bind to different locations on the HbS molecule. In the end, two- to three-residue peptides featuring the aromatic amino acids tryptophan and phenylalanine [121,122] were equivalent in inhibitory activity to peptides containing six residues lacking aromatic amino acids [127]. The concentrations at which aggregation inhibition was observed were, however, too high, pointing to the need for more extensive peptide design. Longer peptides targeting the β4Thr–β’73Asp interaction in addition to the β6Val–β’Phe85/β’Leu88 contact for disruption achieved significant improvement in potency. Moreover, the successful design of the highly potent Peptide II with peptide/hemoglobin inhibition ratio of 0.25:1 brings peptide inhibitors within the potency range needed for clinical relevance [143]. We believe that as structural information (e.g., from high resolution crystallographic analysis or NMR spectroscopy) become more available, it will be possible to properly categorize different peptide inhibitors based on hemoglobin interaction and, in turn, to improve them.

## 5. Benefits and Challenges Associated with Peptide-Based Drugs

Peptide systems, short peptides in particular, have already been employed as potential inhibitors of protein aggregation in a number of pathological conditions involving pathological protein aggregation [123,124,125,144,145,146]. The advantages associated with the use of short peptides include low overall toxicity resulting from the compatibility of peptide inhibitors with living tissues as opposed to small molecule inhibitors. Secondly, metabolic degradation of peptide inhibitors does not yield toxic metabolites, which, combined with the earlier point, allows for a well-tolerated and safe therapeutic option. Furthermore, the high chemical diversity, selectivity, and potency associated with peptide-based inhibitors are versatile, making them viable start-off points in drug discovery campaigns. With regard to protein aggregation in particular, peptide inhibitors, because of their chemical and structural composition, can offer good fits capable of interacting with protein surfaces sufficiently large to disrupt the process of protein aggregation [120]. In spite of these benefits associated with the use of peptides in therapeutics, it should be noted that they are often associated with poor pharmacokinetics relating to short half-life and low oral bioavailability [147,148]. Because of the presence of peptidases, peptide drugs are rapidly degraded and cleared in different body compartments, leading to insufficient exposure of the target system to the administered drug. Available approaches for handling these challenges include the use of D-amino acids or non-natural residues, chemical modifications such as protecting the terminals with appropriate chemical groups (e.g., acetylating the N-terminal and amidating the C-terminal), cyclization, and incorporation of organic molecules in the peptide side chains [149,150,151,152]. Since these approaches alter the physicochemical attributes of the peptide, they can also be useful in improving the membrane partitioning of the peptide drugs. In practice, peptide penetration across cellular barriers has been accomplished via the incorporation of groups facilitating membrane crossing, like positively charged amino acids [153,154,155] or ligands (e.g., sugars), for recognition of membrane receptors [156]. The latter approach has been successfully employed to improve both the stability and the intestinal absorption of peptide drugs [157,158,159]. In the area of cancer drug delivery, where peptide-based chemotherapeutic agents are routinely required to be delivered to intracellular targets, increasing levels of success are being recorded with the development of innovative techniques like the use of cell-penetrating peptides, viral based-vectors, and nanoparticle-based systems [160,161,162]. It is expected that these new developments can be leveraged upon in delivering peptide-based HbS inhibitors into the intracellular compartment of RBCs.

## 6. Conclusions

In this review, we have presented a number of peptide-based inhibitors that have been investigated in relation to their HbS polymerization inhibitory activities. In order to understand their mode of action, we first described the structure of hemoglobin and the inter-residue interactions that drive the polymerization of HbS. Moreover, to put the current knowledge about peptide-based inhibitors into context of other recent drug discovery approaches, we provided a very short review of the most promising of these projects. Here, voxelotor (GBT440) should be emphasized, as it has recently received FDA approval for the treatment of SCD. It is a small molecule that covalently binds to HbS, causing an increase of the proportion of oxy-HbS within RBCs and thereby reducing polymerization as oxy-Hb cannot participate in polymerization. The mode of action of the presented peptide-based inhibitors, on the other hand, are thought to either target the primary interaction between the pathological βVal6 from one HbS molecule and the hydrophobic acceptor pocket in the region of β’Phe85 and β’Leu88 of another HbS molecule or to interfere with one of the various secondary contacts promoting HbS polymerization. It should be mentioned that many of the reported antisickling peptide-based agents were reported more than 30 years ago. Though considering that they were not further developed since then, there is vast room for improving them. The fact that anti-polymerization activity was observed with peptide lengths as short as two amino acids in some cases is, in particular, a significant advantage, since this should permit modifications and design of more potent peptide-based inhibitors for SCD treatment. Indeed, the larger and optimized peptides Hb-B10 [126] and Peptide II (Figure 9) [143] demonstrated a much improved HbS polymerization inhibition over the short peptides that were studied 30−40 years ago. A similar pattern is seen for Alzheimer’s disease, involving the discovery of the first amyloid-aggregation inhibiting D-peptide developed more than 15 years ago [124]. Continuous improvement in the lead D-peptide inhibitor has resulted in a candidate molecule now in a clinical trial [125,163], demonstrating that this line of research is worth pursuing. The advantages of peptide-based compounds outweigh the disadvantages associated with the use of amino acids-based inhibitors, partly because it is possible to circumvent some of them (e.g., by amino acid configuration inversion to increase biological half-life [149,150,151,152]). In conclusion, the level of success reported in the design of peptide inhibitors of protein aggregation should stimulate new investigations into the therapeutic potentials of antisickling peptides for the treatment of SCD. Such peptides must, however, be able to inhibit HbS polymerization at therapeutically relevant concentrations of the peptide inhibitors.

## Figures and Tables

**Table 1:** Short peptides with the best demonstrated inhibitory activity identified in [121,122], given as the minimal inhibitor mole ratio (MIMR) of peptide to HbS necessary to prevent HbS polymerization. The values are means ± standard error. “Suc” stands for succinyl: –OOC–(CH_2_)_2_–CO–.

Peptide	MIMR
Suc-(L-Phe)-(L-Phe)-(L-Phe)	9.5 ± 0.5
Suc-(L-Phe)-(L-Phe)-(L-Phe)-(L-Arg)	10.0 ± 1.0
Suc-(L-Trp)-(L-Trp)	10.0 ± 0.5
Suc-(L-Trp)-(L-Phe)	12.5 ± 0.5
